# Supplementary material for: Prospection of Psychrotrophic Filamentous Fungi Isolated from the High Andean Paramo Region of Northern Ecuador: Enzymatic Activity and Molecular Identification
Source: Microorganisms. 2022 Jan 26;10(2):282. doi: 10.3390/microorganisms10020282 (PMC8880075; doi:10.3390/microorganisms10020282)
Supplement: Supplementary file 1 [file microorganisms-10-00282-s001.zip › microorganisms-1463805-supplementary.pdf]

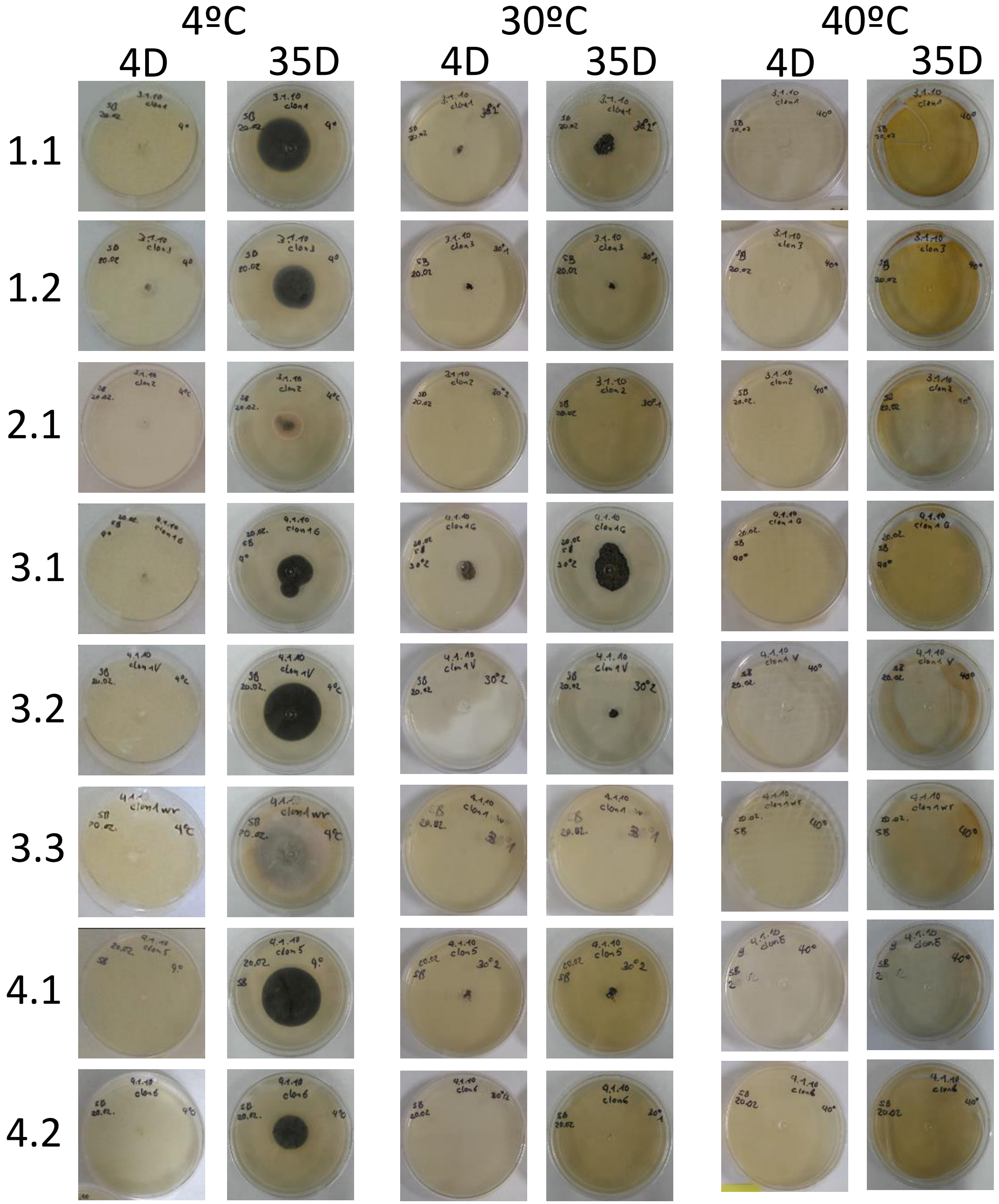

Supplementary Figure S1: Growth of 8 different isolated fungal strains from 4 different sampling sites after 4 and 35 days (D) at different temperatures.

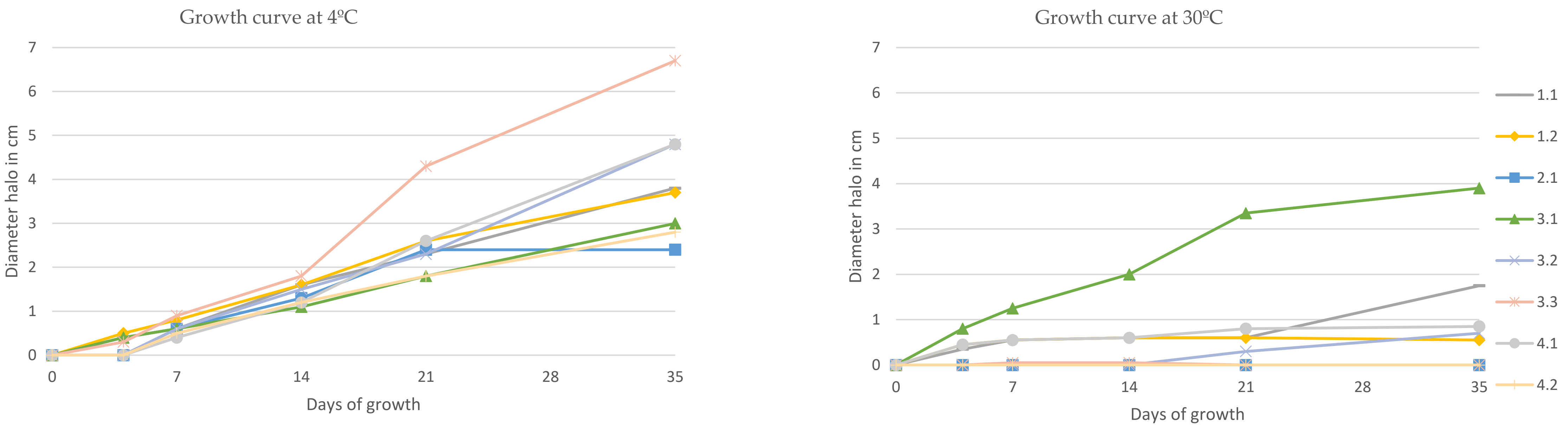

Supplementary Figure S2. Growth curves of isolated fungal strains at 4ºC and 30ºC.

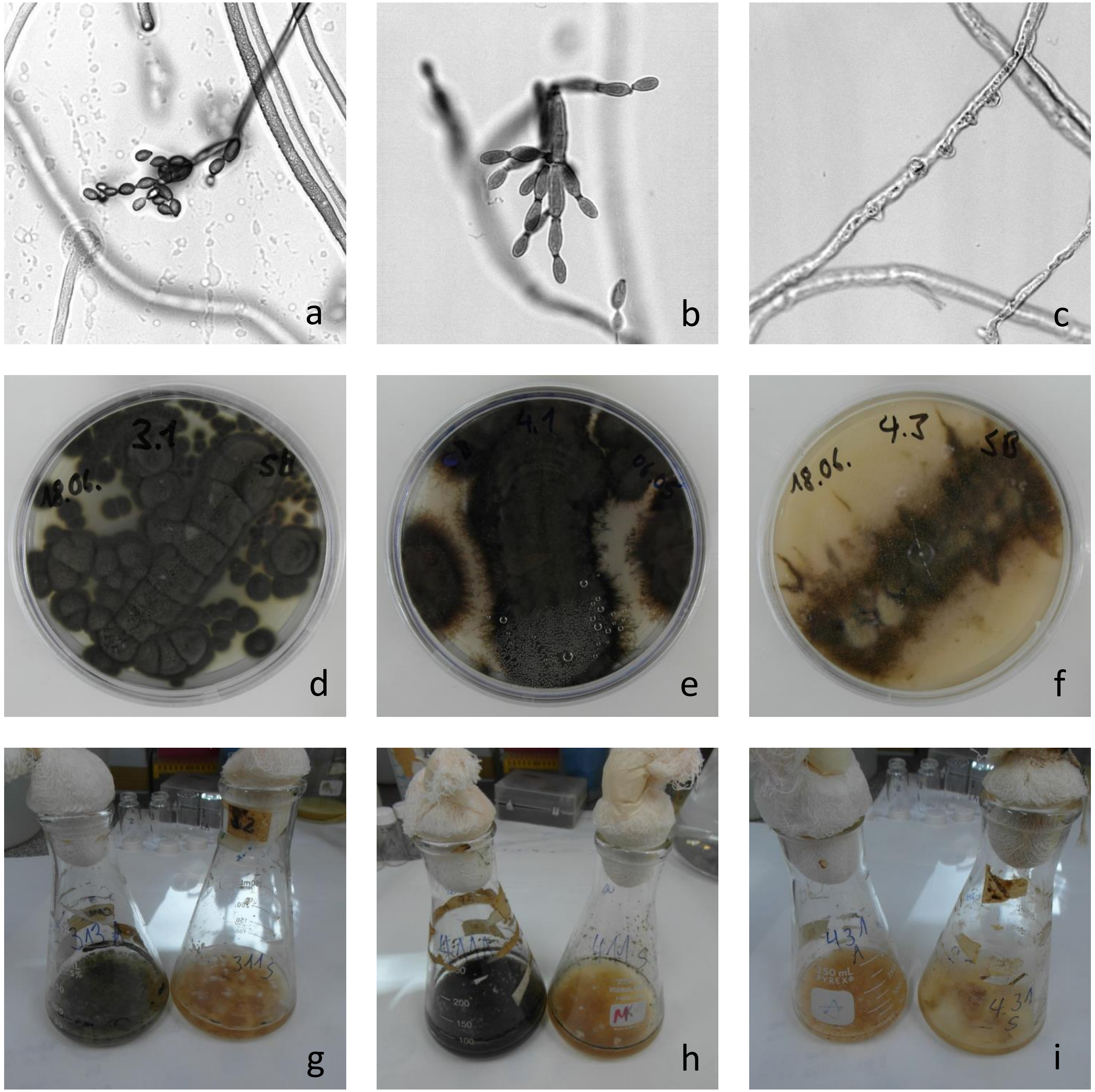

Supplementary Figure S3: Selected and identified fungi, from left to right *Cladosporium michoacanense* (a, d, g), *Cladosporium* sp. (*cladosporioides* species complex)(b, e, h), *Didymella* sp. (c, f, i); top microscopy of sporangium and micelia (a-c), middle row cultures on Potato Dextrose Agar (d-f) and bottom row comparison of shaken culture versus static culture (g-i).

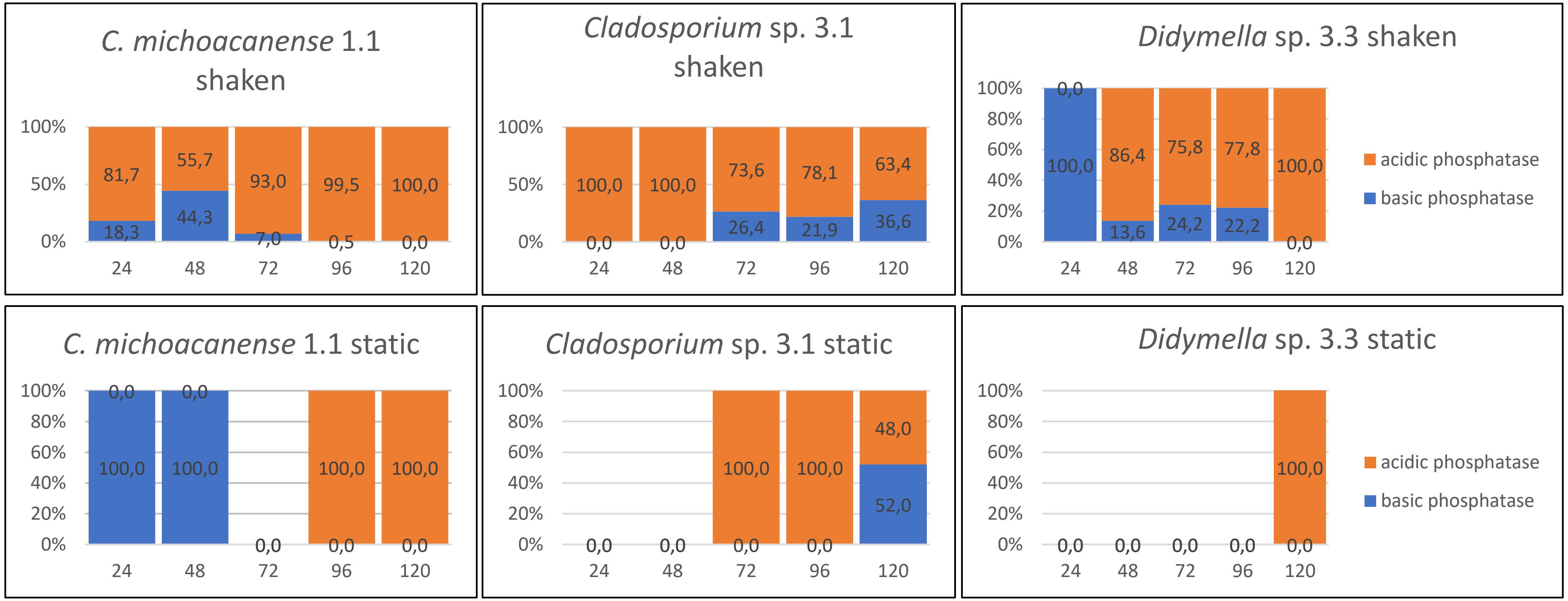

Supplementary Figure S4: Relative phosphate activity over time comparing acidic phosphatase versus alkaline phosphatase

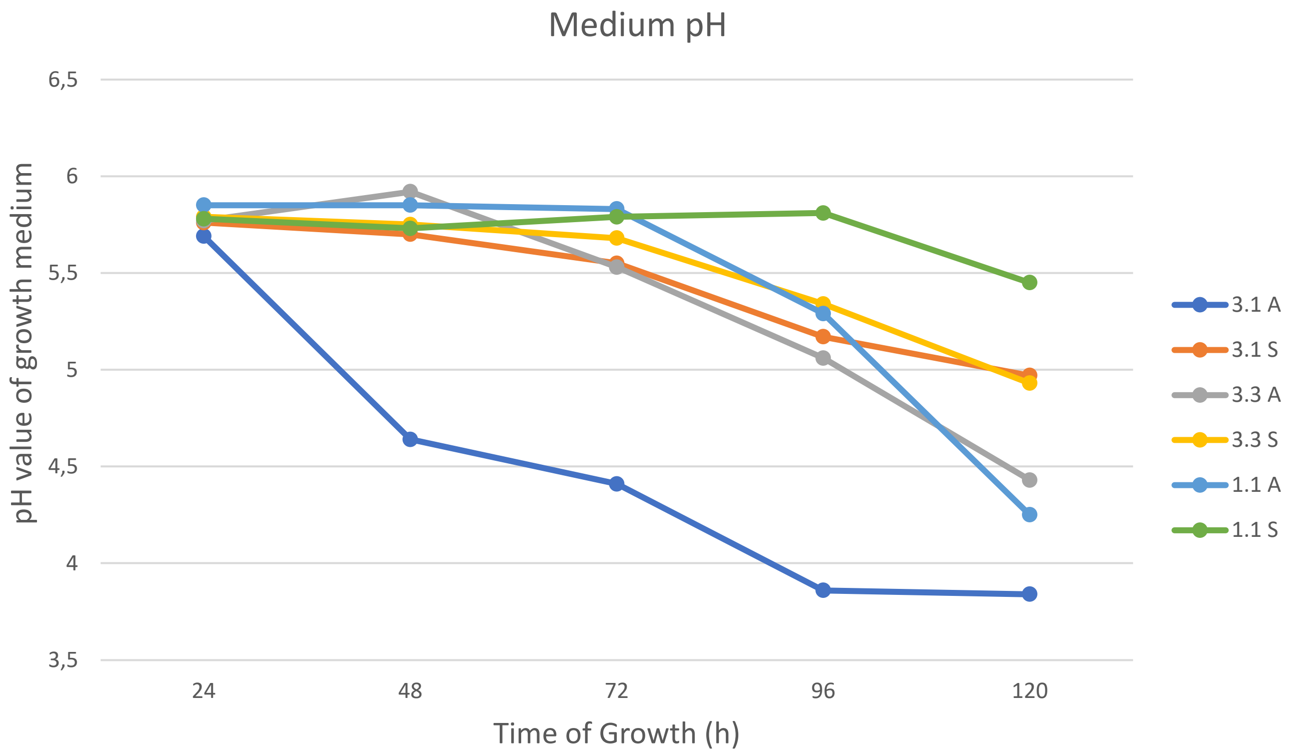

Supplementary Figure S5: Development of growth medium pH over time in cultures under agitation (A) and static (S) conditions for strain 1.1, 3.1 and 3.3

Supplementary Table S1: Sampling site characteristics

Sampling site	Coordinates	Altitude	Temperature (Ø)	Predominant vegetation	N° of chosen isolates
1	Sº 78.701786; Wº 0.631899	3986	9.966	Poaceae. Asteraceae. Verbenaceae. Rosaceae	2
2	Sº 78.702426; Wº 0.631899	3998			1
3	Sº 78.706805; Wº 0.636392	4145	9.552	Poaceae. Apiaceae. Rosaceae	3
4	Sº 78.706972; Wº 0.63616	4149			2
